# Influence of Socioeconomic Factors and Nutritional Advice on Diet Quality in Women of Reproductive Age: A FIGO-DQS Assessment

**DOI:** 10.3390/nu16223855

**Published:** 2024-11-11

**Authors:** Andreea-Maria Mitran, Alina Delia Popa, Andreea Gherasim, Otilia Niță, Graur Mariana, Lidia Iuliana Arhire, Laura Mihalache, Cornelia Mircea, Nikolic Mihaela, Oana Cioancă

**Affiliations:** 1 Faculty of Pharmacy, “Grigore T. Popa” University of Medicine and Pharmacy, 700115 Iasi, Romania; mitran.andreea-maria@email.umfiasi.ro (A.-M.M.); cornelia.mircea@umfiasi.ro (C.M.); oana.cioanca@umfiasi.ro (O.C.); 2 Internal Medicine II Department, Faculty of Medicine, “Grigore T. Popa” University of Medicine and Pharmacy, 700115 Iasi, Romania; andreea.gherasim@umfiasi.ro (A.G.); otilia.nita@umfiasi.ro (O.N.); lidia.graur@umfiasi.ro (L.I.A.); laura.mihalache@umfiasi.ro (L.M.); 3 Faculty of Medicine and Biological Sciences, University “Ștefan cel Mare” of Suceava, 720229 Suceava, Romania; graur.mariana@gmail.com; 4 Faculty of Food and Animal Sciences, University of Life Sciences “Ion Ionescu De La Brad”, 700490 Iasi, Romania

**Keywords:** diet quality, nutritional knowledge, food choices, health care providers

## Abstract

Background/Objectives: Nutritional knowledge, attitudes, and practice are important ways of assuring a healthy pregnancy start, which can be shaped during the pre-pregnancy period by the intervention of healthcare providers. In this cross-sectional study, the main objective was to explore contextual determinants of diet quality in women of fertile age, including socioeconomic factors and sources of information. Methods: Data on socioeconomic background, sources of nutritional advice, and determinants of food choices were collected through an anonymous web-based questionnaire completed by 465 women. Diet quality was assessed with the International Federation of Gynaecology and Obstetrics diet quality score (FIGO-DQS). Results: Better food choices (OR = 1.73; 95% CI: 1.16–2.51), higher knowledge level (OR = 1.66; 95% CI: 1.119–2.466), and healthcare advice (OR = 1.70, 95% CI: 1.119–2.466) increased the chances of having a better diet. Both healthcare providers (β = 0.503, *p* = 0.018) and determinants of food choices (β = 0.520, *p* = 0.011) had a significant influence on the FIGO score, although the advice provided by the healthcare professionals did not yield a significant influence on the determinants of food choices (β = 0.310, *p* = 0.125); Conclusions: Knowledge had a mediator effect on the impact of healthcare guidance on nutrition practices, but it does not fully explain the adherence to healthy lifestyle choices.

## 1. Introduction

A woman’s nutritional status before conception shapes the initial stages of embryonic development, in addition to ensuring maternal health [1]. Nutritional literacy, comprised of both knowledge and practice of dietary guidelines, emerges as one of the best ways of assuring a healthy pregnancy start. Equipping young women with nutrition knowledge and promoting healthy dietary practices during their reproductive years could have far-reaching implications for public health.

Although knowledge alone may not be enough to impose behavioural change, it is a prerequisite [2]. Young women’s understanding and practice of nutrition becomes imperative as they navigate subsequent life stages. This is especially true regarding the transition from preconception to pregnancy, which often occurs unknowingly [3]. As such, ensuring optimal transition into pregnancy requires the acquisition of accurate knowledge and adequate evidence-based practices, starting with the reproductive period. Furthermore, by fostering a favourable food environment for their future families, maternal nutrition literacy provides the groundwork for the development of healthy dietary attitudes, thereby shaping the behaviour of their children [4,5].

In recent years, the preconception period caught on as an opportune phase to get ahead of global issues such as non-communicable diseases [6]. The issue of preconception nutrition has also been addressed through a series of papers, which illustrate the link between preconception nutritional status and pregnancy and birth outcomes and the reduced efficiency of pregnancy interventions in mitigating the adverse effects of pre-pregnancy risk behaviours [7]. Offering accurate and tailored advice empowers women to make informed decisions about their lifestyles and helps dispel misconceptions and myths surrounding nutrition. Preconception nutrition interventions in South Asia led to improved length and a reduction in stunting and wasting [8], while nutrition education interventions have proven useful in initiating behaviour change in women of reproductive age [9]. Nutrition knowledge was also identified as a determinant of pregnancy supplementation with folic acid and multivitamins [10]. Therefore, addressing childbearing-age women’s nutrition, rather than postponing interventions until pregnancy, might be a more gratifying approach.

In Romania, 49% of women of reproductive age are overweight, with 10.3% having obesity. In comparison, the European Union mean is 44.6% and 15.8% [11]. To avert the perpetuation of nutrition-related complications in the next generations, effective interventions are needed. The first step in doing so is identifying the areas that need improvement so that future tailored interventions can target these shortcomings. Some studies have addressed women’s knowledge during preconception, but more data are needed. Given that there is currently no research on nutritional knowledge and practice of reproductive-aged women of Romania, the current study aims to fill this gap in the literature. Additionally, by drawing attention to this subject, this paper intends to provide new insights that could help build targeted healthcare practices and nutrition interventions, ultimately improving women’s health. Furthermore, while the involvement of socioeconomic determinants in nutritional status was extensively explored, healthcare professionals’ interventions on attitudes and practices regarding healthy eating have remained a subject of interest for research [12]. Accordingly, this study aimed to achieve two objectives: (i) assess the relationship between nutrition knowledge, determinants of food choices, and the diet quality of women of reproductive age, and (ii) explore contextual determinants of the aforementioned aspects, including socioeconomic factors and sources of information.

## 2. Materials and Methods

### 2.1. Study Design

We conducted a cross-sectional study on a convenience sample of 465 women of childbearing age who agreed to take part in this study. An original survey was designed to evaluate women’s knowledge of nutrition, as well as their nutrition information sources. We included in our sample healthy non-pregnant women aged between 18 and 50 years who consented to their participation. Exclusion criteria were pregnancy, age outside the reproductive period, and denial of participation. Women who did not complete the entire questionnaire were taken into account when calculating the refusal rate and excluded from the final analysis.

### 2.2. Sample Size Estimation

Sample size calculation was undertaken using the single proportion formula [13]. We selected a 95% confidence level and an assumed prevalence of 50% due to the lack of prior data in our region. It is the single proportion formula (*n* = [Np(1 − p)]/[(d^2^/Z^2^_1−α/2_*(N − 1) + p*(1 − p)]) at 95% confidence interval, where Z^2^_1−α/2_ = 1.96, p (estimated proportion) = 50%, and d (level of precision) = 5% of marginal error [14]. Owing to the lack of other available data, we used the number of women 15–59 years old in Iaşi County reported on 1 July 2017 (N = 231,227) to define the size of the reference group [15]. Based on these calculations, the minimum sample size required was 384. A total of 500 women were invited to participate in the study. The first question generated upon opening the form link assessed the participant’s consent to take part in this study. Only upon consenting to be included in this study did the rest of the questionnaire open. From the final analysis, 35 questionnaires were excluded, representing dropouts (refusals after the initial enrollment in the study or incomplete questionnaires). The rate of refusal was relatively low, totalling 7% of all the women invited to participate in the study.

### 2.3. Data Collection

Data were collected through an anonymous web-based questionnaire. The form completed by the participants included items regarding socio-demographical data, nutritional knowledge, determinants of food choices, and diet quality.

Socio-demographical data were recorded by asking participants about age, area of residence, marital status, education, income, and employment. Participants were also asked about their preferred sources of advice regarding healthy nutrition: social media, relatives, or healthcare professionals.

Nutritional knowledge was assessed through questions regarding participants’ awareness of the recommended servings of main food groups (meat, milk and dairy products, fruits and vegetables, packaged foods) and their knowledge about the importance of iodised salt and healthy weight gain during pregnancy. The participants completed the number of servings or kilograms that they considered correct. Their answers were then coded into two categories, receiving 0 points for incorrect answers and 1 point for correct responses according to the local guidelines’ recommendations [16].

Determinants of food choices were assessed with a short questionnaire that evaluated the main reasons for food selection: nutrient value, price, convenience (easy to prepare), mood, and craving. The positive answers receive 0 points and the negative ones 1 point, except for the question regarding nutrient value, resulting in a score ranging between 0 to 5, a higher score representing a less influenced food choice by emotion or convenience.

Diet quality was assessed with the International Federation of Gynaecology and Obstetrics (FIGO) diet quality score (FIGO-DQS) [17], which is similar to a short FFQ and is composed of 6 food groups: meat and poultry, fruits and vegetables, fish, dairy products, whole grain carbohydrates, and packaged foods. The adequate intake for each group is scored 1 point and the inadequate intake 0 points. The FIGO-DQS score can range between 0 and 6, with higher scores representing a better diet quality. FIGO-NRS (overall nutritional status) includes 3 additional items (oral nutrient supplementation with folic acid, regular sun exposure, and haemoglobin value above 11 g/dL). Thus, the final FIGO-NRS can range between 0 and 9 points.

The total duration of completing the form varied between 25–40 min.

### 2.4. Statistical Analysis

Statistical analysis was carried out using R studio program version 4.4.2. We used the packages ‘psych’ and ‘factoextra’ for principal component analysis and packages ‘mediation’ and ‘boot’ for mediation analysis. Descriptive tests for continuous variables assessed the mean, median, standard deviation, and standard error of the mean. The quantitative variables were checked for normality using the Shapiro–Wilk test. The scores were not normally distributed; thus, the Spearman correlation was used to assess the relationships between the variables. Factor analysis, specifically principal component analysis with varimax rotation, was utilised to construct the socioeconomic index and to identify the principal patterns of food choices. The suitability of the factor analysis for the variables was assessed using the Kaiser–Meyer–Olkin (KMO) test and the Bartlett test. A KMO score greater than 0.5 is considered acceptable, as recommended by Kaiser. In the Bartlett test, significance was set at *p* < 0.05, indicating that the data were suitable for factor analysis. Per the Kaiser criterion, only components with eigenvalues greater than 1 were considered significant [18]. Factor loadings above 0.30 were deemed significant, as recommended by Hair et al. [19].

Associations between socio-demographic factors, food choice determinants, the level of nutritional knowledge, the sources of nutritional advice, and the FIGO-NRS median score were evaluated using the Chi-square test (χ^2^) and binomial logistic regression. Adjusted odds ratios (aORs) and 95% confidence intervals (95% CIs) were determined in multivariate logistic regression models. For all analyses, a *p*-value < 0.05 was considered significant. Multicollinearity was examined using variance inflation factor (VIF) values using the ‘car’ package.

Indirect pathways and mediation effects were tested using the mediation model described by Preacher and Hayes [20]. Significance testing relied on bootstrapping to generate robust bias-corrected confidence intervals for indirect effects and to adjust for potentially non-normal residuals [21].

### 2.5. Ethics

The current study was conducted following the Declaration of Helsinki and approved by the ethics committee of “Gr. T. Popa” Medicine and Pharmacy University (348/28.09.2023). Informed consent was obtained from all subjects involved in the study.

## 3. Results

### 3.1. Socio-Demographic Characteristics of the Sample and FIGO-NRS Score Results

The analysed sample included 465 women of fertile age, with a mean age of 31.76 years old (SD = 8.74), with 77.2% from urban areas. The convenience sample used is not representative of the general population of Iasi County. Statistical analysis revealed significant differences between the age distribution of the sample and that of the general population (18–15 years: population 13.89% vs. sample 27.5%; 25–40 years: 55.80% vs. sample 50.3%; over 40 years: 30.31% vs. sample 22.2%; Chi-square = 16.03, *p* < 0.001), indicating an over-representation of women aged 25–40 years where fertility is at its highest. In contrast, the marital status structure is similar to the general population. The proportion of unmarried women in the sample is 44.7% compared with 42.4% in the general population, and the proportion of married women is 55.3% compared with 57.6% [15]. The Chi-square test (Chi-square = 1.14, *p* = 0.29) shows that there are no significant differences between the two distributions in this respect. Although the sample size is statistically adequate, lack of representativeness remains an important limitation. The average income per capita was 3908 RON, with a standard deviation of 2107.50 RON. This indicates a wide distribution of income among the respondents.

The average number of years of formal education was approximately 15.22 years, with a standard deviation of 2.39 years. This reflects the fact that most respondents had higher education (undergraduate or postgraduate) (Table 1). Most women who participated in this study were married (55.3%) and worked full-time (69.2%). Surprisingly, few women reported following advice from relatives regarding their diet (12.9%). Furthermore, a higher percentage of participants (40.9%) declared social media as a source of diet advice, and only 30.8% declared that they followed the recommendations of healthcare providers. Price (63.4%) and convenience (74.6%) were determinants of food choices for most of the participants, as well as mood (88%) and craving (52%). Nutrient value was important in the decision of food selection for a smaller part of women (53.5%). We found a low level of knowledge regarding certain aspects of nutrition, such as food servings, salt iodisation, or the recommended weight gain during pregnancy.

The mean score for FIGO-NRS is 3.69 (SD = 1.568), with a median of 4. The minimum score is 0, and the maximum is 8. The scores did not have a normal distribution (skewness = 0.176; Kurtosis = −0.555) (Table 2).

Adequate intakes of fruits and vegetables (13.3%), fish (22.2%), and milk and milk products (40%) were achieved by a small number of participants. In contrast, the majority of women met the recommendations for meat and poultry consumption (89%). The recommendations for consumption of these foods, which include 2–3 servings/day of fruits and vegetables, 1–2 servings/week of fish, and daily intake of dairy products, have already been presented in Table 2, as measured by the FIGO score.

### 3.2. Socioeconomic Status Index Construction (SES Index)

The SES index was constructed using socio-demographic characteristics frequently used to distinguish the vulnerable populational groups (age, area of residence, formal education, marital status, income, and employment).

Socioeconomic status is a multifaceted concept used in public health to depict different populations and to identify vulnerable groups. SES could be characterised by different aspects that vary across countries. The Romanian National Institute of Statistics included in its annual report different aspects to characterise the economic and social development of our country, such as gender, marital status, formal education, income, employment, area of residence, and living conditions [22], which were included in our analysis. Usually, these aspects are analysed as distinct variables concerning various health outcomes, such as chronic diseases.

However, a single indicator to assess SES may be more useful for capturing its influence on different health-related outcomes and would permit better control for confounding than modelling different aspects separately [23]. The construction of an SES index based on a set of indicators using principal component analysis (PCA) and varimax rotation to achieve orthogonality was proposed by Moez et al. [23] In our study, the indicators included were formal education, income, marital status, employment status, and age. Age has been included in the social index due to the well-documented risks associated with pregnancy at extreme ages, in particular, under 18 and over 35 years of age. These age groups are associated with obstetric complications and adverse outcomes for both mother and foetus, justifying the integration of age as a key determinant in social risk assessment.

The KMO global score of 0.79 confirmed the adequacy of the data. The principal confirmatory analysis identified two main factors that account for 73.5% of the variance. Principal Component 1 (PC1) included the variables income, education level, and employment, while Principal Component 2 (PC2) was influenced by the civil status component of the index (Table 3). The SES index was created by averaging the PCA scores of the two retained components [23].

### 3.3. Food Choices Determinants

We found a significant correlation between the determinants of food choices score and the individual items of the scale (price: r = 0.605; craving: r = 0.612; mood: r = 0.347; convenience: r = 0.537; nutrient value: r = 0.303), which was tested to assess construct validity, with a value of Cronbach’s alpha of 0.755.

Price, convenience, and mood were important drivers of food choices in our sample. Nutritional value was a criterion in selecting foods only for 53.5% of women, while mood was important for 88%. The KMO factor adequacy coefficient of 0.53 indicated that the identified model can be used for our data. The first component that explains 29% of the variance (PC1) shows positive factor loadings for consumer characteristics associated with direct responsiveness and positive immediate feelings associated with food products (nutritional value, convenience, and mood). Moreover, they reflect the emotional response towards the desired foods (mood) or their convenience (Table 3).

The second component (PC2) was called Unawareness because of the important negative loadings for price and craving. This component reflects the negative feelings associated with cravings and the lack of concerns related to the healthy aspects of food (Table 3). This component could represent consumer concerns or critical viewpoints on the health and satisfaction aspects of their consumption experiences. The mean score of this scale is 2.78 (SD = 0.96). The median value is 3.0. Shapiro–Wilk *p* < 0.001 showed a non-Gaussian distribution.

### 3.4. Knowledge Evaluation

We found a significant correlation between the knowledge score and the individual items of the scale (meat: r = 0.598; fruits and vegetables: r = 0.698; milk: r = 0.663; packaged food: r = 0.300; recommended weight gain during pregnancy: r = 0.325; iodised salt: r = 0.423), which was tested to assess construct validity. Internal fidelity was adequate, with a Cronbach’s alpha value of 0.729.

The KMO factor adequacy coefficient of 0.53 indicated that the identified model can be used for our data. The principal component analysis identified three main components accounting for 59.6% of the variance. The first component grouped items related to knowledge regarding food portions of milk, fruit, and vegetables. The second component explained 18.9% of the variance in grouped items related to meat, packaged food, and the agreement with the salt iodisation (Table 3). The mean score of the knowledge scale is 5.2 (SD = 1.38), with a median of 5. Shapiro–Wilk *p* < 0.001 showed a non-Gaussian distribution.

### 3.5. Relationships Between FIGO-NRS Score, SES Index, Determinants of Food Choices, Knowledge, and Sources of Advice

We found significant correlations between the FIGO-NRS score and knowledge score (Spearman’s rho = 0.174, *p* < 0.001) and determinants of food choices (Spearman’s rho = 0.202, *p* < 0.001). A weak but significant correlation was found between the FIGO-NRS score and the SES index (Spearman’s rho = 0.091, *p* = 0.049). Furthermore, the SES index score was related to both the knowledge level (Spearman’s rho = 0.132, *p* = 0.004) and the score on the determinants of food choices scale (Spearman’s rho = 0.149, *p* = 0.001). We did not find a significant relationship between the knowledge level and the determinants of food choices (Spearman’s rho= −0.044, *p* = 0.348).

We did not find any differences between the proportion of women with FIGO-NRS scores below and above the median according to their socioeconomic status (*p* = 0.472). A significantly higher proportion of women who adhere to better food choices (37.5% vs. 25.8%) had a nutritional score above the median (*p* = 0.006). A similar association was found between the level of nutrition knowledge and the diet quality (37.10% vs. 26.2%, *p* = 0.012). Women who adhered to the diet advice provided by healthcare professionals had an increased probability of having a FIGO-NRS score above the median compared with those who did not follow the nutritional recommendations given by healthcare providers (39.9% vs. 28%, *p* = 0.011). A significant correlation was observed between the percentage of women who utilised social media as a source of nutrition information and those who did not. Concerning the FIGO-NRS score, 26.3% of women who used social media compared to 35.3% who did not use social media had a diet quality score that was higher than the median, *p* = 0.041. We did not find a significant association between the use of diet advice from relatives and diet quality (21.7% vs. 33.1%, *p* = 0.076) (Table 4).

### 3.6. Predictors of FIGO-NRS Score

Better food choices (OR = 1.73; 95% CI: 1.16–2.51), higher knowledge level (OR = 1.66; 95% CI: 1.119–2.466), and healthcare advice (OR = 1.70, 95% CI: 1.119–2.466) increased the chances of having a better diet. In the univariate model, using social media as a source of nutritional advice was associated with lower odds of having a FIGO-NRS score above the median (OR = 0.655; 95% CI: 0.436–0.984). Advice from relatives was not significantly related to the FIGO score in the univariate model (Table 5).

In the multivariate logistic regression model, significant predictors of FIGO-NRS score above the median were food choices (OR = 1.523; 95% CI: 1.004–2.309), healthcare advice (OR = 1.681; 95% CI: 1.085–2.605), and a level of nutritional knowledge above the median (OR = 1.661; 95% CI: 1.119–2.466) (Table 6).

### 3.7. Mediation Analysis of Determinants of FIGO-NRS Score

A mediation analysis was conducted to explore whether advice from healthcare providers mediates the relationship between determinants of food choices and FIGO-NRS scores. The determinants of food choices did not significantly influence the healthcare advice provided by professionals (Path a: β = 0.310, *p* = 0.125). However, both healthcare advice (Path b: β = 0.503, *p* = 0.018) and food choices (Path c: β = 0.520, *p* = 0.010) had a significant direct effect on the FIGO score (Table 7).

The mediation analysis using nonparametric bootstrapping showed a significant total effect of food choices and healthcare advice on the FIGO score (total effect = 0.117, *p* = 0.004), primarily driven by a direct effect of healthcare advice (ADE = 0.110, *p* = 0.006). However, the indirect effect of food choices through healthcare advice was not significant, as the proportion mediated was nonsignificant for both the control group (Prop. Mediated = 0.050, *p* = 0.226) and the treated group (Prop. Mediated = 0.059, *p* = 0.226). We conclude that healthcare advice did not mediate the relationship between food choices and FIGO scores in this sample.

We also examined the hypothesis that the impact of healthcare providers on the FIGO-NRS score is mediated by the level of nutritional knowledge.

A significant positive effect of healthcare providers on the level of nutritional knowledge (β = 0.767, *p* < 0.001) (Path a) was found. This indicates that individuals who followed the healthcare advice were more likely to have better median knowledge compared to those without healthcare guidance. Furthermore, both the level of knowledge and professional nutritional recommendations were significant predictors of FIGO-NRS scores (Paths b and c). Specifically, a higher level of knowledge was related to an increased probability of being in a higher FIGO-NRS group (β = 0.436, *p* = 0.033). Additionally, having access to healthcare guidance was also linked to an increased likelihood (β = 0.459, *p* = 0.032) of better diet quality (Table 8).

**Figure 1:**
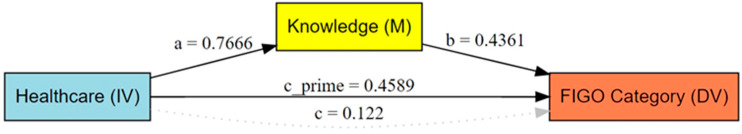
Nutrition knowledge as a mediator of healthcare professionals’ advice effect on FIGO scores.

The mediation analysis assessed the indirect influence of healthcare advice about nutrition on FIGO-NRS by examining the mediating role of knowledge level. The indirect effect of healthcare professionals on FIGO-NRS through the level of knowledge is significant (*p* = 0.044), with an average causal mediation effect (ACME) of 0.021, providing that healthcare access indirectly increases the probability of being above the median in the FIGO score through improved nutrition knowledge. The direct effect of healthcare guidance on FIGO-NRS is statistically significant (*p* = 0.044), with an average estimate of 0.100 (ADE). This indicates that access to healthcare directly enhances the likelihood of having a diet quality above the median. The total effect of professional nutritional advice on the FIGO-NRS score is significant (*p* = 0.012), with an estimate of 0.122. The proportion of the total effect that was mediated through the level of knowledge is not statistically significant (*p* = 0.056), with an average estimate of 0.17 (Table 7, Figure 1).

This analysis indicates that healthcare guidance significantly improves nutritional practice as measured by the FIGO-NRS, both directly and indirectly, with improved nutrition knowledge. The mediation analysis confirmed that a portion of the effect of healthcare providers’ advice on nutrition is mediated by nutrition knowledge, highlighting the importance of educational interventions in improving health outcomes. However, the proportion of the effect mediated by nutrition knowledge was not statistically significant. These data suggest that while nutrition knowledge is an important mediator, other factors may also play a role in the relationship between healthcare advice and nutritional status.

## 4. Discussion

Several key pregnancy risk factors, including micronutrient deficiencies, poor dietary habits, and inadequate maternal body mass index, may require several months of intervention to correct. Given the high prevalence of unplanned pregnancies [24], the critical developmental period during the first few weeks often passes without the mother’s awareness. Consequently, implementing lifestyle changes and dietary recommendations after the onset of pregnancy may be insufficient. From a public health perspective, the most effective strategy for ensuring a healthy start to pregnancy and preventing complications is likely through the implementation of efficient preconception care.

To effectively implement public health strategies tailored to this specific population group, a comprehensive assessment of the current state of nutritional awareness and adherence to healthy eating guidelines is essential. This approach promotes interventions that are not only relevant but also address the population’s specific needs and challenges, increasing the likelihood of successful outcomes in health promotion and disease prevention.

Recently, a nutrition screening tool was developed by the International Federation of Gynaecology and Obstetrics (FIGO) Adolescent, Preconception, and Maternal Nutrition Working Group [25]. The checklist is tailored for both pregnancy and the preconception period, with a maximum score of 9. The mean score for the FIGO checklist was 3.69 (SD = 1.568), with a median of 4. The minimum score was 0, and the maximum was 8. This indicates that all the women in the sample have at least one nutritional risk factor. This finding is in line with other studies that used the FIGO checklist and found that 95% of the participants had at least one risk factor [17,26]. In this sample, we observed poor adherence to several guideline recommendations. Few of the participants had an adequate intake of fruits and vegetables (13.3%), fish (22.2%), and milk and dairy (40%). Most women met the intake of meat and poultry according to the recommended number of servings (89%).

The observed poor intake of fruits and vegetables among reproductive-aged women is in line with the European trends, where only 12% of the population consumed the recommended daily portions. However, the intake among this sample is higher than the national intake of 2% observed in Romania, the lowest among all European countries [27]. This might be attributed to increased health awareness during childbearing years and greater exposure to health education through various resources.

Fish consumption is important in ensuring adequate intake of essential fatty acids, which the body cannot synthesise and must be obtained through diet [28]. Current guidelines recommend an intake of at least two portions of fish per week [16]. First-trimester essential fatty acids levels were associated with significant increases in foetal growth, underscoring the importance of early targeting of adequate intake [29].

Few other studies have employed the FIGO-NRS checklist in women of reproductive age. This is likely attributable to the FIGO checklist being a relatively recent addition to the nutritional screening tools available to practitioners. However, Soepnel et al. have implemented the FIGO nutrition checklist during preconception with similar results [30]. They have also found low levels of adherence to the guidelines regarding fruits and vegetables, fish, and dairy, with high adherence to meat and poultry recommendations. Similarly, 97.4% of participants reported at least one risk factor [29]. A different study, which included pregnant women from Romania, found similar FIGO score results, with poor intakes of fish and whole grains but a higher adherence to fruit, vegetables, and dairy [31].

Most participants (40.9%) declared social media as their preferred source of diet advice, with only 30.8% declaring that they follow healthcare providers’ recommendations. This high reliance on online sources for dietary advice is concerning, as studies have shown that online nutrition content is often low-quality and inaccurate [32]. A significant correlation was also observed between the FIGO-NRS score and social media usage. Women who did not use social media as a source of information had a diet quality score that was higher than the median. These results show a seemingly contradictory effect of using social media as a source of nutrition information. This may be explained by several factors. Social media is a frequent source of conflicting or incorrect nutrition information. Although women use these platforms to access information, much of it may not come from licensed or scientifically validated sources, which could lead to suboptimal dietary decisions [33]. In addition, on social networks, dietary behaviours are often influenced by trends or popularity rather than evidence-based recommendations. This can be misleading and can prevent the adoption of healthy habits [34]. Excessive exposure to different types of nutritional information can make it difficult for users to select what is both relevant and correct, which can negatively affect diet quality. These factors could explain why the percentage of women who used social networks for nutrition information and achieved a diet score above the median is lower compared to those who did not use social networks.

Concerning drivers of food choices, mood (88%) and convenience (74.6%) were declared by most of the participants, as well as price (63.4%) and craving (52%). Nutrient value was important in the decision of food selection for a smaller part of women (53.5%). The mood being reported as the most frequent driver of food choices highlights the need to consider emotional well-being and stress levels when trying to improve dietary behaviours. Moreover, research has also shown that those who consider mood a motive for food choice are more likely to exhibit a positive attitude towards personalised nutrition interventions [35]. However, people for whom convenience and price are the main drivers of food choices might hold less favourable attitudes toward this type of intervention [35]. Overall, these findings suggest that while emotional and practical factors significantly influence food choices, there is potential to more effectively incorporate nutritional value into decision-making processes. Targeted interventions that address the emotional, economic, and sensory determinants of food choices may prove beneficial in encouraging healthier dietary behaviours among this population group.

Overall, low levels of knowledge regarding aspects of nutrition were observed. This raises concerns that such deficiencies in knowledge might lead to poor dietary practices, highlighting the need for better nutrition education interventions. Evidence of this could be the significant correlations between the FIGO-NRS score and knowledge score (Spearman’s rho = 0.174, *p* < 0.001) observed in this sample. This statistically significant relationship indicates that as nutritional knowledge increases, so does the adherence to better nutritional practices as measured by the FIGO-NRS. Although knowledge alone is not sufficient to determine behaviour change, the modest but significant correlation suggests that improving nutritional knowledge could have a meaningful effect on dietary behaviour.

A weak but significant correlation was also found between the FIGO-NRS score and the SES index (Spearman’s rho = 0.091, *p* = 0.049). Furthermore, the SES index score was related to the knowledge level (Spearman’s rho = 0.132, *p* = 0.004), supporting the literature that shows the role of factors such as age, education, and socioeconomic status concerning nutritional knowledge [36,37].

Women who adhered to diet advice provided by healthcare practitioners also had an increased probability of having a FIGO score above the median compared with those who did not (39.9% vs. 28%, *p* = 0.011). In both univariate and multivariate models, better drivers of food choices, higher knowledge levels, and following healthcare dietary advice were predictors of a better FIGO-NRS score. Moreover, mediation analysis showed that knowledge had a mediator effect on the impact of healthcare guidance on nutrition practices, as measured by the FIGO checklist. Altogether, the data indicate that while nutrition knowledge is an important mediator, other factors also play a role in the relationship between healthcare access and nutritional status. Future research should aim to explore the relationship between the dietary advice offered by general healthcare practitioners and how it impacts nutrition-related behaviours.

Targeting women in their reproductive years could yield large public health returns, allowing for more time to implement behaviour change, as compared to interventions targeted towards pregnancy [6,38]. However, implementing changes during this period requires complex resources [39]. As observed in this sample, multiple factors drive individuals’ food choices, including emotional or environmental factors, as well as socioeconomic or knowledge levels. Through mediation analysis, we observed that knowledge mediates the effect of healthcare guidance on nutrition practices, suggesting that information and education can translate into healthier dietary behaviours. This suggests that only providing general advice may not be sufficient when it comes to public health initiatives and that equipping women with tailored knowledge is crucial for effective behavioural change.

One of the main limitations of this study is the high percentage of participants from urban areas with higher education. Although the web-based form of the questionnaire relied on participants’ access to the Internet, online questionnaires can ensure the participants with a level of anonymity that might make them more likely to be honest and offer accurate responses as compared with direct interviews. Another advantage of this approach consists of increased accessibility and convenience, ensuring that participants can complete the survey at their convenience from any location and during any time interval, making it easier to target broader groups of people. Even though some previous studies have investigated the nutrition practice [40] and knowledge [10] of pregnant women, this is, to the best of the author’s knowledge, the first study to investigate the nutrition practice and knowledge of reproductive-aged women in Romania. Moreover, as the questionnaires were completed by the participants rather than through direct interviews, the completion process relied on the participants’ level of literacy and the voluntary participation of individuals who proactively chose to complete the questionnaire.

In our study, we identified important associations between knowledge, healthcare guidance, and dietary practices, but the cross-sectional design does not make it possible to conclude cause-and-effect relationships. Future research should build upon these findings by investigating in greater detail how environmental factors shape dietary behaviours in this particular population group. This could provide a broader understanding of the social factors that impact nutritional awareness and practices among reproductive-aged women.

## 5. Conclusions

Poor adherence to dietary guidelines was observed in this sample of reproductive-aged women. Specifically, alarmingly low intakes of fruits, vegetables, and fish servings were observed. The median score for FIGO-NRS was 4, with all the women (100%) presenting at least one high-risk dietary practice. Most of the participants reported mood (88%) and convenience (74%) as drivers of food choices and social media (40.9) and healthcare practitioners (30.8) as sources of nutrition information. In both univariate and multivariate models, better drivers of food choices, higher knowledge levels, and the ability to follow healthcare dietary advice were predictors of a better FIGO-NRS score. Moreover, mediation analysis showed that knowledge had a mediator effect on the impact of healthcare guidance on nutrition practices, as measured by the FIGO checklist. Although nutritional knowledge is an important instrument through which healthcare professionals can influence the quality of women’s diets, it does not fully explain the adherence to healthy lifestyle choices. These results highlight the need for public health interventions targeted at women during their reproductive period, tailored to their specific needs and unique behaviour drivers.

## Figures and Tables

**Table 1:** Sample characteristics and survey responses.

Socioeconomic Characteristics		Frequency	Percentage
Age (years old)	18–25	128	27.5
	25–40	234	50.3
	>40	103	22.2
Formal education (years of formal education)	≤8	126	27.1
	8–12	210	45.2
	>12	129	27.7
Income (RON)	<1500	76	16.3
	1500–3000	85	18.3
	3000–5000	148	31.8
	>5000	156	33.5
Employment	no	120	25.8
	part-time	23	4.9
	full time	322	69.2
Civil status	single	100	21.5
	relation	108	23.2
	married	257	55.3
Food Choices Reasons			
Price	No	170	36.6
	Yes	295	63.4
Craving	No	223	48
	Yes	242	52
Convenience	No	118	25.4
	Yes	347	74.6
Mood	No	56	12
	Yes	409	88
Nutritional value	No	216	46.5
	Yes	249	53.5
Nutrition Advice Source			
Relatives	No	405	87.1
	Yes	60	12.9
Healthcare professionals	No	322	69.2
	Yes	143	30.8
Social media	No	275	59.1
	Yes	190	40.9
Nutrition Knowledge			
Fruits and vegetables	No	225	48.4
	Yes	240	51.6
Milk and dairy	No	126	27.1
	Yes	339	72.9
Snack	No	24	5.2
	Yes	441	94.8
Meat	No	196	42.2
	Yes	269	57.8
Iodised salt	No	232	49.9
	Yes	233	50.1
Recommended gestational weight gain	No	334	71.8
	Yes	131	28.2

**Table 2:** FIGO-NRS score—descriptive characteristics.

Item	Positive Answers	
	Counts	% of Total
Fruits and vegetables (2–3/day)	62	13.3
Meat and poultry (2–3/week)	414	89
Fish (1–2/week)	103	22.2
Milk and diary (daily)	186	40
Whole grain products (≥1/day)	231	49.7
Packaged foods (5/week)	441	94.8
Folic acid supplements	366	78.7
Regular sun exposure	238	51.2
Hb > 11 g/dL	324	69.7

**Table 3:** Index loadings and variance explained (principal component analysis).

Variable/ Statistic	SES Index		Variable/ Statistic	Food Choices Determinants		Variable/ Statistic	Knowledge		
	PC1	PC2		PC1	PC2		PC1	PC2	PC3
Age	0.406	0.340	Price	0.176	0.682	Meat servings knowledge	0.371	−0.497	0.282
Education	0.438	−0.474	Craving	0.189	0.647	Milk and dairy servings knowledge	0.636	0.208	0.066
Income	0.511	−0.238	Convenience	0.622	−0.053	Fruits and vegetables knowledge	0.589	0.347	−0.062
Employed	0.516	−0.131	Mood	0.350	−0.291	Packaged food knowledge	0.331	−0.441	−0.275
Married	0.338	0.764	Nutritional Value	0.650	−0.165	Iodised salt knowledge	0.008	0.514	−0.487
-	-	-	-	-	-	Weight gain during pregnancy	0.022	−0.358	−0.773
Standard Deviation	1.688	0.910	Standard Deviation	1.205	1.079	Standard deviation	1.201	1.065	0.909
Proportion of Variance	0.570	0.165	Proportion of Variance	0.290	0.233	Proportion of Variance	0.240	0.189	0.166
Cumulative Proportion	0.570	0.735	Cumulative Proportion	0.290	0.523	Cumulative Proportion	0.240	0.429	0.596

**Table 4:** Associations between socioeconomic status, food choice determinants, knowledge, source of nutritional advice, and diet quality.

		FIGO-NRS Median Score		χ^2^	*p*
		Above Median	Below or Equal Median		
SES index median	≤median	71 (30.10%)	165 (69.90%)	0.518	0.472
	>median	76 (33.20%)	153 (66.8%)		
Food choices determinants (median score)	≤median	60 (25.80%)	173 (74.20%)	7.422	0.006
	>median	87 (37.50%)	145 (62.50%)		
Knowledge (median score)	≤median	61 (26.20%)	172 (73.80%)	6.375	0.012
	>median	86 (37.10%)	146 (62.90%)		
Relatives	No	134 (33.10%)	271 (66.90%)	3.152	0.076
	Yes	13 (21.70%)	47 (78.30%)		
Healthcare	No	90 (28.00%)	232 (72.00%)	6.497	0.011
	Yes	57 (39.90%)	86 (60.10%)		
Social media	No	97 (35.3%)	178 (64.70%)	4.170	0.041
	Yes	50 (26.3%)	140 (73.70%)		

**Table 5:** Determinants of FIGO-NRS score.

Univariate Binary Logistic Regression	B	S.E.	Sig.	OR	95% CI for OR	
					Lower	Upper
SES index > median score	0.144	0.2	0.472	1.154	0.781	1.707
Food choices > median score	0.548	0.202	0.007	1.73	1.164	2.571
Knowledge > median score	0.507	0.202	0.012	1.661	1.119	2.466
Healthcare providers (yes)	0.536	0.211	0.011	1.709	1.129	2.584
Relatives(yes)	−0.581	0.331	0.079	0.559	0.293	1.07
Social media (yes)	−0.423	0.208	0.042	0.655	0.436	0.984

**Table 6:** Predictors of FIGO-NRS score.

Multivariate Binary Logistic Regression	B	S.E.	Sig.	OR	95% CI for OR	
					Lower	Upper
SES index > median score	−0.007	0.208	0.973	0.993	0.661	1.493
Food choices > median score	0.42	0.212	0.048	1.523	1.004	2.309
Knowledge > median score	0.427	0.208	0.04	1.532	1.019	2.304
Healthcare providers (yes)	0.519	0.223	0.02	1.681	1.085	2.605
Relatives (yes)	−0.506	0.353	0.152	0.603	0.302	1.205
Social media (yes)	−0.364	0.215	0.09	0.695	0.456	1.059

A score above the median indicates a food choice less influenced by emotion or convenience factors such as cravings or ease of preparation.

**Table 7:** Mediation effect of healthcare nutritional advice on the relationship between food choices and FIGO-NRS score.

Term		Estimate	Std. Error	z Value	Pr > (|z|)
Path a	(Intercept)	−0.0994	0.11159	−0.891	0.373
	Food choices	0.3100	0.20183	1.536	0.125
Paths b and c	(Intercept)	−1.209	0.165	−7.308	0.000
	Healthcare	0.503	0.213	2.363	0.018
	Food choices	0.520	0.203	2.557	0.010
Mediation analysis	Effect	Estimate	95% CI Lower	95% CI Upper	*p*-value
	ACME (control)	0.006	−0.004	0.02	0.222
	ACME (treated)	0.007	−0.004	0.03	0.222
	ADE (control)	0.109	0.024	0.19	0.006
	ADE (treated)	0.110	0.024	0.19	0.006
	Total effect	0.117	0.035	0.20	0.004
	Prop. Mediated (control)	0.050	−0.042	0.31	0.226
	Prop. Mediated (treated)	0.059	−0.051	0.34	0.226
	ACME (average)	0.006	−0.003	0.02	0.222
	ADE (average)	0.110	0.024	0.19	0.006
	Prop. Mediated (average)	0.054	−0.048	0.33	0.226

Treated—food choices unrelated to emotion or convenience; control—food choices related to convenience. A score above the median indicates a food choice less influenced by emotion or convenience factors such as cravings or ease of preparation.

**Table 8:** Mediation effect of nutritional knowledge on the relationship between healthcare providers’ advice and FIGO-NRS.

	Term	Estimate	Std. Error	Z Value	Pr > (|z|)
Path a	(Intercept)	−0.2371	0.1122	−2.113	0.03
	Healthcare	0.7666	0.2063	3.715	0.00
Path b and c	(Intercept)	−1.1495	0.1600	−7.184	0.00
	Knowledge (median)	0.4361	0.2052	2.125	0.033
	Healthcare	0.4589	0.2149	2.135	0.032
Mediation analysis	Effect	Estimate	95% CI Lower	95% CI Upper	*p*-value
	ACME (control)	0.020	0.0005	0.04	0.044
	ACME (treated)	0.023	0.0006	0.04	0.044
	ADE (control)	0.099	0.005	0.19	0.044
	ADE (treated)	0.102	0.005	0.20	0.044
	Total Effect	0.122	0.024	0.21	0.012
	Prop. Mediated (control)	0.165	−0.001	0.61	0.056
	Prop. Mediated (treated)	0.192	−0.002	0.63	0.056
	ACME (average)	0.021	0.0006	0.04	0.044
	ADE (average)	0.100	0.005	0.20	0.044
	Prop. Mediated (average)	0.178	−0.001	0.62	0.056

ACME (average causal mediation effect) is the average of the mediation effect among the control (not receiving nutrition counselling) and treated groups. The term ‘control’ refers to participants who did not receive, and ‘treated’ refers to those who received nutrition advice from health professionals. ACME and ADE estimates were obtained using bootstrapping.

## Data Availability

Data used in this study will be available from the corresponding authors upon request.
